# Results of emergency salvage lung resection after chemo- and/or radiotherapy among patients with lung cancer

**DOI:** 10.1093/icvts/ivac043

**Published:** 2022-03-07

**Authors:** Haruaki Hino, Takahiro Utsumi, Natsumi Maru, Hiroshi Matsui, Yohei Taniguchi, Tomohito Saito, Koji Tsuta, Tomohiro Murakawa

**Affiliations:** 1 Department of Thoracic Surgery, Kansai Medical University, Osaka, Japan; 2 Department of Pathology, Kansai Medical University, Osaka, Japan

**Keywords:** Emergency operation, Salvage lung resection, Chemotherapy, Radiotherapy, Lung cancer

## Abstract

**OBJECTIVES:**

This study aimed to elucidate the outcomes of emergency salvage surgery following life-threatening events (serious haemorrhage and/or infections) among patients with lung cancer who had undergone chemo- and/or radiotherapy.

**Materials and Methods:**

We analysed the data of patient from 2015 to 2020, retrospectively. The clinical characteristics, including preoperative treatment, perioperative outcomes and survival time, were analysed.

**RESULTS:**

Of the 862 patients who underwent primary lung cancer surgeries, 10 (1.2%) underwent emergency surgeries. The preoperative clinical characteristics were: median age, 63.7 years [interquartile range (IQR) 55–70.5]; sex (male/female), 9/1; clinical staging before initial treatment (I/II/III/IV), 1/1/3/5; initial treatment (chemoradiotherapy/chemotherapy/proton beam therapy), 5/4/1; and indications for emergency surgery (lung abscess/lung abscess with haemoptysis/haemoptysis/empyema), 5/3/1/1. The selected procedures and results were as follows: lobectomy/bilobectomy/pneumonectomy, 8/1/1 (all open thoracotomies); median operation time, 191.0 min (IQR 151–279); median blood loss, 1071.5 ml (IQR 540–1691.5); postoperative severe complications, 3 (30%); hospital mortality, none; median postoperative hospital stay, 37 days (12–125); control of infection and/or haemoptysis, all the cases; final outcome (alive/dead), 3/7 (all the cancer deaths); median postoperative survival, 9.4 months (IQR 4.3–20.4); and median survival from initial treatment, 19.4 months (IQR 8.0–66.9).

**CONCLUSIONS:**

Emergency salvage lung resection is a technically challenging procedure; however, the results were feasible and acceptable when the surgical indication, procedure and optimal timing were considered carefully by a multidisciplinary team. Although the aim was palliation, some patients who received additional chemotherapy afterwards and, thus, had additional survival time.

**Figure:**
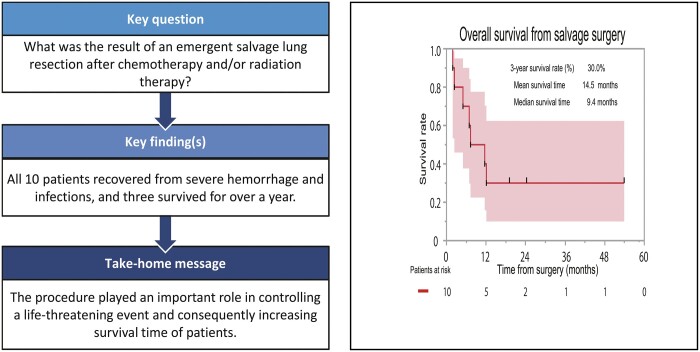

## INTRODUCTION

Lung cancer is the leading cause of cancer-related deaths worldwide, including Japan [1]. Generally, patients with advanced lung cancer (clinical stage III and IV) undergo definitive chemoradiotherapy; however, a local recurrence rate of between 28.1% and 34.1% was detected [2]. Similarly, among early-stage lung cancer patients treated with high-dose radiotherapy due to inoperability, a local recurrence rate of 0–67.0% was reported, which is currently an issue [3]. Recently, general thoracic surgeons demonstrated a rescue surgery targeted for pretreated patients with local recurrence or cancer persistence. The so-called salvage surgery has been performed in those with lung cancer who received chemotherapy and/or radiotherapy and in whom the outcomes of short-term morbidity, mortality and long-term survival were feasible, compared with those who were still receiving chemotherapy [4–9]. According to the variety of salvage surgery series for lung cancer that has been reported thus far, the patient characteristics have been stratified using pretreatment procedures, i.e. definitive chemoradiotherapy, targeted therapy using epidermal growth factor receptor–tyrosine kinase inhibitor (EGFR–TKI), high-dose stereotactic body radiation therapy or carbon ion therapy (CIT) and proton beam therapy (PBT) [10–12]. In investigating the value of elective salvage surgery for complete cancer curability, we encountered some emergency lung cancer patients with a life-threatening event such as a lung abscess from a serious infection, empyema or haemoptysis after/during chemotherapy or chemoradiotherapy or radiotherapy, which were defined differently from the other types of oncological salvage surgeries [13, 14]. In selected conditions, we performed an emergent palliative surgery for patients with a fatal lung cancer who met the criteria; however, we may have missed the chance or optimal timing for emergent rescue surgery. Since such cases are rarely encountered, the significance and impact of emergent salvage lung resection for the purpose of saving lives has not been elucidated, to date. Therefore, this study aimed to evaluate not only the feasibility but also the postoperative survival outcomes of emergent palliative salvage surgery in lung cancer patients with life-threatening haemoptysis and/or infections following chemo- and/or radiotherapy.

## MATERIALS AND METHODS

### Ethics statement

It was conducted in accordance with the Declaration of Helsinki and the Research Review Board at Kansai Medical University, Osaka, approved this study on 22 September 2021 (approval number 2021132). The requirement to obtain informed consent was waived.

This study was conducted retrospectively, using the clinical database of a single institution. The clinical data that were gathered included age, sex, initial treatment, aetiology of emergent condition, operative procedure, amount of bleeding, operative time, preoperative clinical and postoperative pathological stages, histological findings, postoperative complications, survival time and postoperative additional therapy. The inclusion criteria for emergency salvage surgery were as follows: patients who underwent definitive chemoradiotherapy or chemotherapy alone for locally advanced lung cancer or radiotherapy for early-staged lung cancer, as an initial treatment due to inoperable poor general condition or the desire of the patient (excluding those who received induction chemoradiotherapy followed by surgery); patients who had life-threatening complications, which could not be treated medically (e.g. haemoptysis from residual tumour, uncontrolled infection following tumour necrosis, empyema); and those who needed an emergency lung resection. The preoperative indications and optimal timing were discussed in multidisciplinary conferences or short additional meetings. All the included patients were treated previously by the thoracic oncology department of internal medicine and/or radiology department at our institute. Preoperative severe life-threatening events and postoperative complications were obtained retrospectively from a chart view with the latter being defined according to the Clavien–Dindo classification [15]. Overall survival (OS) time was calculated as the time from the date of initial treatment or surgery to the date of death or last follow-up. Preoperative and postoperative tumour staging was determined using the 8th edition of the TNM staging system of the International Union against cancer [16], while the histological tumour type was determined using the 3rd edition of the World Health Organization Classification of Tumors [17]. The pathological response was classified according to the criterion of the 6th edition of the General Rules of Clinical and Pathological Records of Lung Cancer in Japan [4], as follows: effect (Ef.) 0: no pathological response, Ef.1: slight pathologic response (divided into Ef.1a and EF.1b, with viable cancer cells remaining in more than two-thirds and in one-third to two-thirds, respectively, in the resected specimen), Ef.2: moderate response (viable cancer cells remaining in less than one-third in the resected specimen) and Ef.3: complete response (no viable cancer cells in the resected specimen).

### Data availability

The data underlying this article will be shared on reasonable request to the corresponding author.

### Statistical analysis

The continuous data were presented as either the mean and range or median and interquartile range (IQR). The categorical data were expressed as a frequency count and percentage. The survival curves were calculated using the Kaplan–Meier method. The statistical analysis was performed with JMP software ver. 12 (SAS Institute Inc., Cary, NC, USA).

## RESULTS

**Figure 1:**
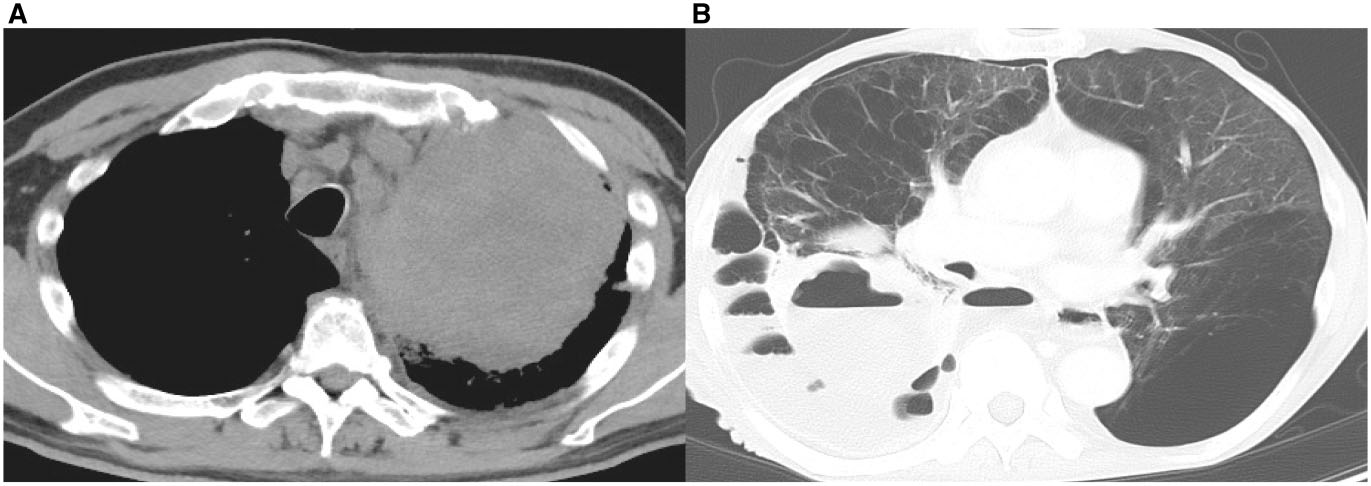
(**A**) Preoperative computed tomography of patient number 5 diagnosed with a huge pleomorphic carcinoma located in the left upper lobe. The tumour changed into a necrotic mass after chemotherapy. (**B**) Preoperative computed tomography of patient number 9 diagnosed with a large squamous cell carcinoma located in the right lower lobe. The tumour was broken and complicated with acute empyema after chemotherapy.

**Figure 2:**
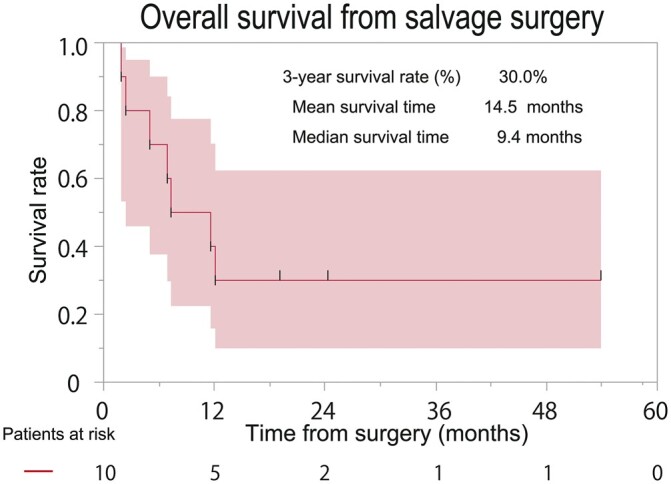
Overall survival curve of 10 patients after salvage surgery.

We analysed the data of the patients retrospectively, from September 2015 to October 2020. A total of 862 patients with primary lung cancer underwent surgery. Among them, we included 10 patients (1.2%) who met the criteria for an emergency salvage lung resection. The preoperative clinical characteristics, including the initial clinical staging and treatment of all 10 patients, are shown in Table 1. The preoperative median age was 63.7 years (IQR: 55.0–70.5), and 9 patients were male. Five patients were diagnosed as stage IV under TNM staging at initial treatment. Four patients received definitive chemoradiotherapy for their initial therapy, 5 received chemotherapy alone and 1 received PBT for early-stage lung cancer. An immune checkpoint inhibitor (ICI) was used for 2 patients prior to surgery. The indications for emergent surgery were as follows: uncontrolled lung abscess [5 out of 10 patients (5/10; 50%)], uncontrolled lung abscess with haemoptysis (3/10; 30%), massive haemoptysis requiring intubation to prevent suffocation (1/10; 10%) [13], and acute empyema (1/10; 10%). While there was no specific predominant organism that caused the lung abscess, *Candida, Actinomyce*s, an aerobic gram-positive rod and *Enterococcus faecalis* were identified as causative organisms in 4 out of 8 patients with a lung abscess formation. The preoperative images of the computed tomography scan in patient number 5 (lung abscess with haemoptysis) and 9 (acute empyema) are shown in Fig. 1. The most common histopathological type of lung cancer observed was squamous cell carcinoma (7/10; 70%). The perioperative surgical results are shown in Table 2. The mean and median operation times were 220.2 and 191.0 min (range: 108–424, IQR: 151–279), respectively, and the mean and median bleeding volumes were 1119.8 and 1071.5 mL (range: 63–2059, IQR: 540–1691.5), respectively; blood transfusions were needed in 8 patients. The operative procedures were as follows: lobectomy (8/10; 80%), bi-lobectomy (1/10; 10%) and pneumonectomy (1/10; 10%); these were all performed using open thoracotomy. Additional procedures included pulmonary artery plasty (1/10; 10%) and bronchial plasty (1/10; 10%). Coverage of the bronchial stump was carried out in 7 patients using a pericardial fat pad (7/10; 70%). Severe postoperative complications as per the Clavien–Dindo classification grade III or higher (i.e. postoperative empyema, pneumonia, chest drainage for haemothorax) were observed in 3 patients (3/10; 30%); however, 30-day postoperative mortality was not observed. The postoperative outcomes are shown in Table 3. The mean and median follow-up periods from the salvage surgery were 14.5 and 9.4 months (range: 1.9–53.9, IQR: 4.3–20.4), respectively, and those from the initial treatment were 37.4 and 19.4 months (range: 5.4–115.3, IQR: 8.0–66.9), respectively. Five patients were diagnosed as pathological stage IV (5/10; 50%) and 1 had no residual tumour. The most common pathological efficacy was confirmed to be Ef.1 (8/10; 80%). Five patients (5/10; 50%) received postoperative treatment including additional chemotherapy for residual tumour and radiotherapy for bone metastasis. The 3-year OS rate from the salvage surgery was 30.0% (95% confidence interval 10.0–62.4%; central image and Fig. 2) and the 3- and 5-year OS rates from initial treatment were both 30.0% (95% confidence interval 10.0–62.4%). At the end of the observation period, 7 patients died of lung cancer from distant organ metastasis (for example to the brain and bone), 1 patient was alive without cancer, another was alive with adrenal metastasis and was treated with chemotherapy and another was alive with cancer and was using EGFR–TKI for pleural dissemination for approximately 2 years with almost complete remission.

**Table 1:** Clinical characteristics of 10 patients who underwent emergent salvage lung cancer surgery

Number	Age	Sex	Pretreatment clinical stage	Initial treatment prior to surgery	Completeness of chemotherapy	Etiology for emergency	Histology
1	77	M	cT2aN2M0-IIIA	Platinum doublet chemotherapy and concurrent RT (60 Gy)	Complete	Lung abscess and haemoptysis	Sq
2	66	M	cT3N2M0-IIIB	Platinum doublet chemotherapy and concurrent RT (60 Gy)	Incomplete	Lung abscess	Sq
3	61	M	cT4N2M1b(OSS)-IVA	Platinum doublet chemotherapy	Incomplete	Lung abscess	LCNEC
4	48	M	cT2aN1M0-IIB	Left upper lobectomy → platinum doublet chemotherapy and concurrent RT (60 Gy) for recurrence	Complete	Lung abscess and haemoptysis	Sq
5	68	M	cT3N2M1b(ADR)-IVA	Platinum doublet chemotherapy including angiogenesis inhibitor and immune checkpoint inhibitor	Complete	Lung abscess	Pleo
6	57	M	cT4N2M1a(EFF)-IVA	Platinum doublet chemotherapy → single agent	Complete	Lung abscess and haemoptysis	Sq
7 (13)	70	M	cT1cN0M0-IA3	Proton beam therapy (60.4 Gy)	Complete	Hemoptysis	Ad
8	69	F	cT4N2M0-IIIB	Platinum doublet chemotherapy and concurrent RT (60 Gy)	Complete	Lung abscess	Sq
9	72	M	cT4N0M1a (EFF)-IVA	Platinum doublet chemotherapy including immune checkpoint inhibitor	Incomplete	Acute empyema	Sq
10	49	M	cT2aN1M1b(OSS)-IVB	Platinum doublet chemotherapy	Incomplete	Lung abscess	Sq

Ad: adenocarcinoma; ADR: adrenal metastasis; EFF: malignant effusion; F: female; LCNEC: large cell neuroendocrine carcinoma; M: male; OSS: bone metastasis; Pleo: pleomorphic carcinoma; RT: radiation therapy; Sq: squamous cell carcinoma.

**Table 2:** Perioperative results of 10 patients who underwent emergency salvage lung cancer surgery

Number	Procedure	Coverage of bronchial stump	Radicality	Interval from initial pretreatment (months)	Interval from last pretreatment (months)	Operation time (min)	Bleeding amount (mL)	BT	Postoperative stay (days)	Complications	Grade	30-Day mortality
1	Open right upper lobectomy	Pedicled pericardial fat pad	R1	16.50	15.07	294	986	Yes	44	Empyema	II	No
2	Open right lower lobectomy	Intercostal muscle and pedicled pericardial fat pad	R0	0.57	0.43	274	1569	Yes	50	Prolonged air leak	II	No
3	Open right lower lobectomy	None	R1	1.37	0.30	154	694	Yes	15	None		No
4	Open left pneumonectomy	Pedicled thymus	R0	5.70	2.90	424	554	Yes	125	Empyema	IIIb	No
5	Open left upper lobectomy	None	R2	12.47	0.87	203	2094	Yes	58	Postoperative haematoma	IIIa	No
6	Open right upper/middle lobectomy and bronchoplasty	Pedicled pericardial fat pad	R2	12.37	0.43	261	1157	No	24	None		No
7 (13)	Open left upper lobectomy and pulmonary artery plasty	Pedicled pericardial fat pad	R2	79.73	0.23	179	498	Yes	31	Subacute interstitial pneumonia	II	No
8	Open left upper lobectomy	None	R0	96.17	93.17	163	2059	Yes	12	None		No
9	Open right lower lobectomy and lavage	Pedicled pericardial fat pad	R1	0.40	0.37	108	1524	Yes	86	Postoperative pneumonia	IV	No
10	Open right upper lobectomy	Pedicled pericardial fat pad	R2	3.57	0.77	142	63	No	25	None		No

BT: blood transfusion.

**Table 3:** Postoperative results of 10 patients who underwent emergency salvage lung cancer surgery

	Age	Sex	Postoperative stage	Pathological stage (8th)	Pathological tumour size (cm)	Pathological efficacy	Postoperative therapy	Final outcomes	Survival from surgery (months)	Survival from initial treatment (months)
1	77	M	ypT2bN0M0	IIA	4.7	ef1a	Immune check point inhibitor	Cancer death	11.6	28.1
2	66	M	ypT2aN1M0	IIB	7	ef1b	No	Alive with another lung cancer	53.9	54.5
3	61	M	ypT4N2M1a (OSS)	IVA	10	ef1a	Platinum doublet	Cancer death	7.3	8.6
4	48	M	No residual tumour	No residual tumour	0	ef3	No	Cancer death	6.9	12.6
5	68	M	ypT4N2M1b (ADR)	IVA	10.5	ef1b	No	Cancer death	1.9	14.4
6	57	M	ypT4N1M0	IIIA	10.7	ef1b	Platinum doublet radiation → immune check point inhibitor	Cancer death	12.1	24.4
7 (13)	70	M	ypT2bN0M1a (PLE)	IVA	4.5	ef1b	EGFR–TKI	Alive with cancer	24.3	104.1
8	69	F	ypT1cN0M0	IA3	2.2	ef1a	No	Alive without cancer	19.1	115.3
9	72	M	ypT4N0M1a (EFF)	IVA	10	ef1a	No	Cancer death	5.0	5.4
10	49	M	ypT1aN0M1b (OSS)	IVA	0.2	ef2	Palliative radiation	Cancer death	2.4	6.0

ADR: adrenal metastasis; EFF: malignant effusion; EGFR–TKI: epidermal growth factor receptor–tyrosine kinase inhibitor; F: female; M: male; OSS: bone metastasis; PLE: pleural dissemination.

## DISCUSSION

In this study, we demonstrated that the short- and long-term results of emergency salvage lung resection in as many as 10 patients were feasible and acceptable. Although the aim of the procedure was palliation, mainly to rescue patients from life-threatening complications such as a lung abscess and/or haemoptysis during or after chemotherapy and/or chemoradiotherapy, the patients were able to recover from these fatal events, and some of them even lived for over a year with additional postoperative chemotherapy. Although the procedure demanded technical skills and adequate decision-making for optimal patient selection with appropriate operative timing, the postoperative results were considered reasonable and permissible, which had a different meaning compared with other types of elective salvage surgery where a patient did meet the optimal operative criteria.

Prior studies regarding the classification of salvage lung cancer surgery have stratified it into 4 categories based on the type of preoperative treatment and condition of the patient [10–12]: category 1, a procedure for lung cancer recurrence or persistence after definitive chemoradiation therapy [4–8, 18, 19, 20]; category 2, surgery for lung cancer recurrence or persistence after high-dose stereotactic body radiation therapy, CIT and PBT for a patient who refused surgery or had inoperable early-stage lung cancer due to multiple comorbidities [4, 8, 9]; category 3, an operation for relapse after targeted therapies with EGFR–TKI or anaplastic lymphoma kinase inhibitor [21, 22]; and category 4, a rescue procedure against an emergent condition caused by serious adverse events of haemoptysis, lung abscess or empyema after chemotherapy and/or radiation therapy different from such as categories 1, 2 and 3 above [11, 12, 23].

To describe and compare those surgical results precisely, the morbidity/mortality/OS from surgery stratified into 3 categories was 14.8–40%/0–11.1%/31–75% for the 5-year OS (category 1) [6, 19, 24–27], 0–25%/0–4.8%/57.7–82% for 3-year OS (category 2) [4, 8, 9, 28] and 5.6–11.1%/0%/75% for 3-year OS or 17 months of the median OS (category 3) [21, 22], respectively. Although category 4 case series were limited, the results of 3 cases were reported with a morbidity/mortality/mean OS rates of 100%/0%/13 ± 5 months, respectively [12].

In this study that included a total of 10 patients, the morbidity/mortality/survival rates were 30%/0%/30% for 3-year OS, which were comparable to prior reports. Unlike the other categories, which were generally performed as elective surgeries with the aim of completely resecting the residual tumour, the main purpose of category 4 was to rescue a symptomatic lung cancer patient with life-threatening complications, regardless of oncological resectability, including stage IV lung cancers. The indication for true emergent and palliative surgery was 5 patients with stage IV and the other 5 patients with stage I–III were indicated for a salvage surgery with urgency. Therefore, our emergency surgery was not considered as a true oncological salvage surgery in line with the other types of salvage surgery. However, some of them (5/10; 50%) underwent postoperative chemotherapy and/or radiotherapy again, which may have contributed additionally to postoperative survival. With regard to the use of a palliative procedure to control the bleeding from a residual tumour other than from the surgery, an embolism for the bronchial artery or intubation of the contralateral airway using the packing of a bronchial orifice with bronchoscopy was the palliative choice. It is especially likely to be performed in a pre-end-stage condition or in a patient with irreversible sequelae such as brain metastasis. In our case series, all 10 patients recovered consequently from these fatal complications, and 5 (50%) patients obtained a chance to receive chemotherapy or radiation therapy again, with the possibility of long-term survival. Moreover, 3 of 5 patients with stage IV cancer (3/5; 60%) could resume postoperative chemotherapy or radiation therapy again and survive for 1.9, 2.4 and 7.3 months after the emergency salvage surgery. In addition, 3 of 5 patients in stages other than clinical IV (3/5; 60%) survived for over a year with or without lung cancer. It was reported that postoperative chemotherapy and/or radiation after salvage surgery improved their survival, which supported our results [23]. However, since we only had 10 cases who required emergent operation, we were not certain whether our procedure had the effect of prolonging the prognosis; larger numbers of similar cases might be encountered in daily clinical practice without being discussed by multidisciplinary teams that include general thoracic surgeons. Considering that effective chemotherapy, such as the use of ICIs, is emerging daily, we expect an increase in the number of patients requiring emergent or elective salvage surgery, which may provide the value of surgery. Ideally, surgeons should be cautious in managing these patients and should recommend the option of elective or emergency salvage lung resection only on a case-by-case basis, after discussions with a multidisciplinary team (consisting of the oncologist, radiologist and general thoracic surgeon) in daily clinical conferences; this might increase the chances of saving the life of a pretreated advanced lung cancer patient [6, 19].

With regard to the preoperative clinical characteristics of our emergent salvage series, the clinical stage IV at initial treatment, a histology of squamous cell carcinoma with a large tumour size and short interval from the last date of chemotherapy and/or radiation therapy accounted for the majority compared with those who had elective salvage surgery of categories 1, 2 and 3. Comparing the perioperative characteristics, an open thoracotomy, significant intraoperative bleeding, postoperative pathological status of Ef.1 and 2, non-down-staged case and R2 resection were the clinical features of our 10 emergency cases. With regard to the perioperative clinical course among these cases, we were implemented cautious airway management in patients who experienced more or less haemoptyses. In patient number 7, with massive preoperative haemoptysis, a double lumen tube was inserted in the contralateral side to protect the patient against massive blood during respiration. However, prior to the emergent surgery, the massive amount of haemoptysis was stopped transiently, the patient was placed in a right recumbent position and the thoracotomy was performed without problems and consequently, the condition of the contralateral lung was maintained after surgery. Since 8 of the 10 patients undergoing chemotherapy experienced complications such as serious inflammatory and infectious conditions just before surgery, the extent of intraoperative bleeding was higher than those who had elective surgery; this was due to resections with fresh adhesion. Intraoperatively, we resected these fresh adhesions in almost all the cases, and massive bleeding from the thoracic wall was observed. We managed the haemostasis for those bleeding from the thoracic wall surface with gauze packing at the time of each procedure, and the haemostasis was performed cautiously. Consequently, although blood transfusions were needed in most cases, we did not perform any re-operations for the postoperative bleeding. The toxicity or side effects of the chemotherapy were not evident after the surgery. These observations implied that a serious adverse event may occur frequently among patients with large-sized squamous cell lung carcinoma who undergo effective chemo- or chemoradiotherapy such as the use of ICIs. We first considered the use of percutaneous drainage for the lung abscess; however, the patient was in a septic condition and percutaneous drainage was ineffective, since some of the abscess content drained spontaneously through the bronchi and pleura or the abscess content was not fluid that could be drained easily. Therefore, we removed the tumour itself, which was the origin of the infection and this was considered a better approach to rescue the fatal septic condition. Under those patient conditions, we should be cautious in managing patients with life-threatening complications and consider the optimal timing for salvage surgery.

Considering the indication and timing of an emergency salvage lung resection, there have, thus far, been no definite recommendations from prior studies. Based on our experience from these 10 cases, a relatively younger generation, good performance status and activity of daily life before being complicated with severe adverse events were considered as optimal indications for emergency salvage surgery. Those with remnants of cancerous cells located in a single lobe, regardless of distant organ metastasis, were also acceptable candidates for timely salvage surgery. In case 4, we performed a pneumonectomy because the patient was young, age 48 years, and was able to tolerate a pneumonectomy. Furthermore, a lesion of tumour necrosis was located in the central side of the hilar structure without any distant organ metastasis. Therefore, we performed a pneumonectomy to control the infection and lung cancer, although this type of procedure was suitable for a single lobectomy, like most of our cases. With regard to the optimal timing of emergency salvage surgery, it was completely different from the oncological timing of other types of elective salvage surgery. Theoretically, these indications were consistent with surgery for infectious diseases such as aspergilloma or nontuberculous mycobacteriosis, for the purpose of controlling these life-threatening infections [29]. New effective chemotherapeutic agents for lung cancer have been developed recently and 4 of the 10 patients (4/10; 40%) in this case series were receiving chemotherapeutic agents (ICIs) prior to or after the salvage surgery [30]. Since a greater number of good responses are being reported in patients with advanced lung cancer, candidates for salvage surgery are expected to increase in future.

With regard to the prognostic indices among the recipients of salvage lung cancer surgery after chemoradiotherapy, a simple lobectomy (versus complex resection), macroscopic and microscopic complete resection (versus R1 and R2 resection; microscopic and macroscopic tumour remnants), pathological non-lymph node metastasis (versus positive lymph node metastasis) and down-staged condition (versus unchanged or upstaged condition) were the significantly favourable prognostic factors for long-term survival (category 1) [4, 6, 20]. In this study, 2 of 3 patients whose conditions were down-staged with R0 resection after the use of definitive chemoradiotherapy and chemotherapy, survived for over a year with cancer and cancer-free status, respectively. This suggested that patients who underwent emergency salvage surgery had some common prognostic factors regardless of the different pretreatment agents and conditions. A patient who underwent PBT for stage I lung cancer and emergency salvage lung resection for massive haemoptysis also survived for almost 2 years with pleural dissemination, receiving EGFR–TKI with almost complete remission [13]. This patient demonstrated that the use of additional chemotherapy after emergency salvage surgery contributed to longer survival. Accordingly, newly developed anti-cancer drugs such as ICIs and postoperative chemotherapy may enable longer survival after salvage surgery if the pretreatment clinical staging is less than IV.

### Limitations

This study had some limitations. We had a selection bias for performing emergent salvage surgery, because the previous treatment of the patients and the aetiology of the emergent conditions differed considerably, and the indications for the operation were decided by the surgeons and multidisciplinary team at that time in a single institution. Another limitation was that the results of our emergency salvage surgery were not compared with those who had not had emergency surgery. Moreover, chemotherapeutic agents, including the newly developed ICI, were administered recently in some of the patients, which had substantial effects on lung cancer survival. However, we were able to analyse selected patients who received emergency life-saving surgery for uncontrollable infections or massive bleeding; this allowed for a robust analysis of possibility for emergency salvage lung resection for patients with pretreated lung cancer. In the future, we will conduct a multicentre trial to elucidate the appropriate indication, procedure, optimal timing and the significance of emergency salvage surgery, which will support our result.

## CONCLUSIONS

Emergency salvage lung resection is technically challenging and involves difficulty in decision-making for the optimal procedure and timing for surgery; however, the results of this study were feasible and acceptable. It is also advisable that patient selection, optimal timing and procedures against a life-threatening event are discussed carefully and considered by a multidisciplinary team. Although the aim was palliation, our results suggested that emergent salvage lung resection played an important role in controlling serious haemorrhage and/or infections, and consequently, afforded some patients additional long-term survival. A similar study with a larger sample size should be performed in future; the results of which may demonstrate the significance of the procedure.

## Notes

Presented at the 29th European Conference on General Thoracic Surgery, 20–22 June 2021 (VIRTUAL).

## Abbreviations

**ABBREVIATIONS**
CIT: Carbon ion therapy
EGFR–TKI: Epidermal growth factor receptor–tyrosine kinase inhibitor
ICI: Immune checkpoint inhibitor
IQR: Interquartile range
OS: Overall survival
PBT: Proton beam therapy
